# Effects of combined chromium picolinate and sodium bicarbonate supplementation on growth performance, rumen fermentation, nutrient digestibility, protozoal population, and carcass traits in lambs fed high-concentrate diets

**DOI:** 10.1007/s11250-026-05178-4

**Published:** 2026-06-22

**Authors:** Erkil Onur Günüç, Mustafa Salman

**Affiliations:** 1 Sheep Breeding Research Institute, Feeds and Animal Nutrition Department, Ministry of Agriculture and Forestry, Bandırma, Balıkesir Türkiye; 2 Department of Animal Nutrition and Nutritional Diseases, Ondokuz Mayıs University, Faculty of Veterinary Medicine, Samsun, Türkiye Türkiye

**Keywords:** Chromium picolinate, Sodium bicarbonate, Rumen fermentation, Nutrient digestibility, Protozoal population, Carcass traits, Lambs, High-concentrate diets

## Abstract

High-concentrate feeding systems are widely applied in intensive lamb production to improve growth efficiency; however, such diets may impair rumen stability and nutrient utilization. This study evaluated the individual and combined effects of chromium picolinate (CrPic) and sodium bicarbonate (Bic) supplementation on growth performance, feed efficiency, rumen fermentation characteristics, protozoal population density, nutrient digestibility, and carcass traits in lambs fed high-concentrate diets. Twenty-eight weaned male Bafra lambs were randomly allocated to four dietary treatments (*n* = 7): control, CrPic (0.25 mg elemental chromium/head/day), Bic (1.5% of dietary dry matter), and CrPic + Bic. Following a 15-day adaptation period, lambs were fed experimental diets for 63 days under individual-feeding conditions. Feed intake, body weight, rumen fermentation parameters, and apparent nutrient digestibility were determined. Body weight and dry matter intake were not significantly affected by dietary treatment (*P* > 0.05), although both variables changed over time throughout the experimental period (*P* < 0.001). Average daily gain and feed conversion ratio were significantly influenced by treatment, time, and treatment × time interactions (*P* < 0.05), indicating phase-dependent responses to supplementation. Lambs receiving the combined chromium picolinate and sodium bicarbonate supplementation exhibited improved feed efficiency and greater average daily gain during specific growth phases, particularly between days 14 and 28. Chromium picolinate supplementation reduced ruminal ammonia-N concentration (*P* < 0.001), increased protozoal counts (*P* < 0.001), and improved acid detergent fiber digestibility (*P* < 0.05), whereas sodium bicarbonate supplementation had limited effects on overall performance and rumen fermentation characteristics. Chromium picolinate and sodium bicarbonate exert distinct physiological effects on rumen fermentation, nutrient utilization, and productive performance in lambs fed high-concentrate diets. The combined supplementation strategy improved feed efficiency during specific growth phases, suggesting that its benefits may be greatest during adaptation to intensive feeding systems. However, these responses were not consistently reflected in carcass characteristics.

## Introduction

High-concentrate feeding systems are widely applied in intensive lamb production to accelerate growth rates, shorten finishing periods, and improve economic efficiency (Plaizier et al. 2018; Humer et al. 2018; Zebeli and Metzler-Zebeli 2019). Nevertheless, diets rich in rapidly fermentable carbohydrates impose substantial challenges to rumen homeostasis by increasing acid load, depressing ruminal pH, and altering microbial activity (Patra and Aschenbach 2018; Ding et al. 2020; Carmona et al. 2022). Under such conditions, disturbances in rumen fermentation frequently result in impaired fiber degradation, inefficient nitrogen utilization, and unstable volatile fatty acid (VFA) profiles, ultimately limiting feed conversion efficiency despite adequate dietary energy supply (Hristov et al. 2019; Morgavi et al. 2020; Oliveira et al. 2023).

Among nutritional strategies developed to mitigate ruminal instability, dietary buffering agents particularly sodium bicarbonate have been extensively evaluated in ruminant feeding systems (Zebeli and Metzler-Zebeli 2012; Dong et al. 2019; Gallo et al. 2020). Sodium bicarbonate supplementation increases ruminal buffering capacity, moderates declines in rumen pH, and supports the activity of fibrolytic microorganisms under acidogenic conditions (Penner et al. 2019; Eun et al. 2022). Consequently, improvements in dry matter and fiber digestibility have been consistently reported in ruminants fed high-concentrate diets (Carmona et al. 2022; López et al. 2022). However, the effects of sodium bicarbonate on growth performance and feed efficiency remain inconsistent, suggesting that stabilization of the rumen environment alone may be insufficient to fully optimize productive efficiency under intensive feeding regimes (McCann et al. 2020; Oliveira et al. 2023).

In contrast to buffering strategies that primarily act within the rumen, chromium functions as a metabolic modulator at the post-absorptive level. Trivalent chromium enhances insulin receptor signaling, facilitates cellular glucose uptake, and improves metabolic partitioning of absorbed nutrients (Spears et al. 2017; Vincent 2019; Sun et al. 2021). In ruminants, chromium supplementation has been associated with improved feed efficiency, altered energy metabolism, and enhanced tolerance to metabolic stress, particularly under intensive production conditions (Pechová and Pavlata 2007; Bai et al. 2021; Qiu et al. 2022). Organic chromium sources, such as chromium picolinate, exhibit greater bioavailability than inorganic forms and have been reported to influence both systemic metabolism and ruminal fermentation patterns (Spears 2019; Sano et al. 2019; Hung et al. 2023).

Recent evidence further indicates that chromium supplementation may modify rumen fermentation by increasing propionate production and reducing the acetate-to-propionate ratio, thereby enhancing the supply of glucogenic substrates for hepatic glucose synthesis (Reynolds et al. 2019; Khiaosa-ard and Zebeli 2019; Li et al. 2021). Because propionate is the primary precursor for gluconeogenesis in ruminants, such shifts in VFA profiles are considered metabolically advantageous and may directly support growth efficiency (Patra and Aschenbach 2018; Hristov et al. 2019). However, responses to chromium supplementation remain variable across studies, with some reporting significant improvements in performance and others observing limited or inconsistent effects (Ren et al. 2020; Bai et al. 2023). These inconsistencies suggest that the efficacy of chromium supplementation may depend on upstream rumen conditions that govern fermentative efficiency and substrate availability.

In addition to their effects on rumen fermentation and nutrient utilization, both chromium and buffering agents have been associated with alterations in rumen microbial ecology and carcass development. Changes in protozoal populations may influence nitrogen recycling, microbial protein synthesis, and fiber degradation within the rumen ecosystem. Previous studies have reported that dietary chromium supplementation can modify microbial activity and ruminal fermentation patterns, whereas sodium bicarbonate may indirectly affect protozoal populations by stabilizing ruminal pH under high-concentrate feeding conditions. Furthermore, chromium supplementation has been evaluated for its potential effects on carcass characteristics and tissue deposition in growing lambs, although reported responses have been inconsistent. While some studies have observed improvements in carcass yield and muscle development, others reported minimal or no effects, suggesting that responses may depend on dosage, feeding conditions, and animal physiological status.

High-concentrate feeding systems frequently induce subclinical ruminal instability, which may restrict the metabolic benefits of chromium supplementation by limiting microbial efficiency, propionate supply, and nitrogen utilization (Zebeli and Metzler-Zebeli 2012; Humer et al. 2018; Khiaosa-ard and Zebeli 2019). Consequently, chromium supplementation alone may not fully overcome metabolic constraints imposed by an unstable ruminal environment. Integrating metabolic modulators with dietary strategies that stabilize rumen fermentation may therefore represent a more effective approach for improving nutrient utilization and feed efficiency in intensively fed lambs (Dieho et al. 2017; Morgavi et al. 2020).

The combined use of sodium bicarbonate and chromium picolinate offers a biologically plausible strategy to simultaneously address ruminal and post-absorptive limitations. Sodium bicarbonate may enhance rumen stability and fiber digestion by improving fermentative conditions, whereas chromium picolinate may improve rumen stability, nutrient utilization, and productive performance under intensive feeding conditions (Reynolds et al. 2019; Bai et al. 2021; Qiu et al. 2022). Despite this conceptual complementarity, integrated evaluations of buffering agents and organic chromium supplementation in lambs fed high-concentrate diets remain scarce, particularly under controlled individual-feeding conditions that allow precise assessment of feed intake, digestibility, and rumen fermentation responses (Dong et al. 2019; López et al. 2022).

Therefore, the objective of the present study was to evaluate the individual and combined effects of chromium picolinate and sodium bicarbonate supplementation on growth performance, feed efficiency, rumen fermentation characteristics, protozoal population density, nutrient digestibility, and carcass traits in lambs fed high-concentrate diets. It was hypothesized that dietary supplementation with chromium picolinate and sodium bicarbonate, individually or in combination, may improve rumen stability, nutrient utilization, and productive performance under intensive lamb-fattening conditions. These findings are particularly relevant for lamb production systems in subtropical and semi-intensive regions, where high-concentrate feeding strategies are increasingly adopted to improve productivity and economic efficiency.

## Materials and methods

### Ethical approval

All experimental procedures involving animals were reviewed and approved by the Local Ethics Committee for Animal Experiments of Ondokuz Mayıs University (OMU-HADYEK; Approval No. 2019/43). Animal management, handling, and experimental procedures were conducted in accordance with national regulations for animal welfare and complied with the ARRIVE 2.0 guidelines for reporting animal research.

### Animals, experimental design and housing

A total of twenty-eight clinically healthy weaned male Bafra lambs (10–12 weeks of age) were used in the experiment. Lambs were selected to ensure homogeneity among groups with respect to age, birth type, health status, and initial body weight. Prior to the start of the experiment, all animals underwent routine health examinations, were treated for internal and external parasites, and vaccinated against enterotoxemia.

Lambs were randomly allocated to one of four dietary treatments (*n* = 7 lambs per treatment):

(i) Control (basal diet),
(ii) Chromium picolinate (CrPic),
(iii) Sodium bicarbonate (Bic),
(iv) Chromium picolinate + sodium bicarbonate (CrPic + Bic).

Animals were housed individually in separate pens, allowing precise control of feed intake and individual measurements. Each pen was equipped with an individual feeder and water trough, and fresh drinking water was available ad libitum throughout the experiment. Each lamb represented an independent experimental unit.

The experiment consisted of a 15-day adaptation period followed by a 63-day experimental feeding period, resulting in a total duration of 78 days. Each lamb was considered the experimental unit for all statistical analyses.

### Experimental diets and feeding management

The feeding regimen was designed to reflect an intensive lamb-fattening system based on high-concentrate diets while allowing precise control of dietary supplementation. All experimental diets were formulated on a dry matter (DM) basis to contain 85% concentrate feed and 15% alfalfa hay, consistent with the feeding strategy applied in the doctoral study. Diets were formulated to be isonitrogenous (approximately 16% crude protein) and isocaloric (approximately 2,700 kcal/kg metabolizable energy) to ensure that any observed effects were attributable to dietary additives rather than differences in nutrient density.

The concentrate feed was prepared using standard feed ingredients commonly employed in lamb fattening rations. Prior to the start of the experiment, representative samples of all feed ingredients were analyzed to determine their dry matter and chemical composition. These analyses were used to accurately formulate the experimental diets and to calculate nutrient supply and additive inclusion levels. The ingredient composition of the concentrate diets and the chemical composition of the concentrate feed and alfalfa hay are presented in Tables 1 and 2, respectively.

**Table 1:** Ingredient composition of the experimental concentrate diets (g/kg dry matter)

Ingredient (g/kg DM)	Control	CrPic	Bic	CrPic + Bic
Corn	320.29	320.29	320.29	320.29
Wheat bran	201.24	201.24	201.24	201.24
Sunflower meal	142.47	142.47	142.47	142.47
DDGS	120.00	120.00	115.00	115.00
Wheat	75.00	75.00	75.00	75.00
Barley	60.00	60.00	60.00	60.00
Molasses	50.00	50.00	50.00	50.00
Limestone powder	20.00	20.00	20.00	20.00
Salt	10.00	10.00		
Sodium bicarbonate	–	–	15.0	15.0
Vitamin–mineral premix	1.00	1.00	1.00	1.00
Total	1000	1000	1000	1000

Chromium picolinate was supplemented at 0.25 mg elemental chromium per lamb per day

**Table 2:** Chemical composition of the concentrate feed and alfalfa hay (g/kg dry matter, unless otherwise stated)

Feed Ingredients	Concentrate Feed	Alfalfa Hay
Dry Matter	95.20	91.01
Ash	6.84	8.44
Organic Matter	93.16	91.56
Crude Protein	15.39	14.88
Crude Fat	2.15	1.81
Crude Fiber	6.01	39.96
Nitrogen-Free Extract	69.61	34.91
Neutral Detergent Fiber	27.93	65.77
Acid Detergent Fiber	11.06	55.4
ME, kcal/kg DM	2783	1763

DM: dry matter; OM: organic matter; CP: crude protein; EE: ether extract; NDF: neutral detergent fiber; ADF: acid detergent fiber

Lambs were assigned to one of four dietary treatments: a non-supplemented control diet, a chromium picolinate–supplemented diet (CrPic), a sodium bicarbonate–supplemented diet (Bic), and a combined chromium picolinate and sodium bicarbonate diet (CrPic + Bic).

Chromium picolinate was included in the experimental diets to supply 0.25 mg of elemental chromium per lamb per day (mg/head/day). This supplementation level was selected in accordance with the doctoral experiment and previous studies reporting metabolic responses to organic chromium under intensive feeding conditions. Based on observed dry matter intake, this dose corresponded approximately to 0.30–0.35 mg Cr/kg dietary dry matter. Chromium supplementation was expressed on a per-animal daily basis to ensure consistent chromium intake irrespective of individual variation in feed consumption.

The required amount of chromium picolinate for each lamb was weighed daily using a precision balance and applied individually as a top-dress onto the concentrate feed immediately before feeding. This approach ensured complete ingestion of the supplement and minimized variability in chromium intake among animals.

Sodium bicarbonate was incorporated into the diets at a level of 1.5% of dietary dry matter, as applied in the doctoral study and described in detail in the AFST submission. This inclusion rate was selected to provide effective ruminal buffering capacity under high-concentrate feeding conditions without negatively affecting feed intake or palatability. Expressing sodium bicarbonate inclusion on a dry matter basis allowed precise and reproducible application of the buffering strategy across animals and feeding periods.

Sodium bicarbonate was thoroughly mixed into the concentrate portion of the diet prior to feeding to ensure homogeneous distribution of the buffering agent within the ration.

Lambs were fed ad libitum, with daily feed allowances adjusted to exceed the previous day’s intake by approximately 10%, thereby avoiding unintended feed restriction. Feed was offered in two equal meals per day at 07:30 and 17:30 h. Alfalfa hay was provided as the sole roughage source and offered separately from the concentrate feed.

Individual feed intake was recorded daily for each lamb. The amounts of concentrate and roughage offered were weighed, and refusals were collected and weighed prior to the morning feeding on the following day. Individual dry matter intake was calculated based on the dry matter content of the feeds and refusals. Fresh and clean drinking water was available to all animals throughout the experimental period.

### Growth performance measurements

Body weights of lambs were recorded at the beginning of the experimental period and subsequently on days 14, 28, 42, 56, and 63 of the experiment. All weighings were conducted after approximately 12 h of feed withdrawal to minimize variation due to gut fill. Live weight gain was calculated from the differences between successive body weight measurements.

Daily dry matter intake (DMI) was calculated by subtracting the amount of feed refused from the amount of feed offered. Feed conversion ratio (FCR) was calculated as the ratio of dry matter intake to live weight gain for the corresponding period.

### Apparent nutrient digestibility

Apparent nutrient digestibility was determined using the total fecal collection method. Digestibility trials were conducted between days 49 and 56 of the experimental period. To facilitate fecal collection, individual fecal collection bags were fitted to each lamb.

Feces were collected daily, weighed, and 10% of the total fecal output was sampled and stored at − 20 °C. At the end of the collection period, fecal samples obtained over six consecutive days were pooled on an individual animal basis to form composite samples. Composite fecal samples were dried at 60 °C for 48 h and ground prior to analysis.

Apparent digestibility coefficients of dry matter (DM), organic matter (OM), crude protein (CP), neutral detergent fiber (NDF), and acid detergent fiber (ADF) were calculated using standard equations based on nutrient intake and fecal excretion.

### Rumen fermentation parameters

Rumen fluid samples were collected from each lamb during the final week of the experiment, 2 h after the morning feeding, using a rumen probe. Immediately after collection, rumen pH was measured using a calibrated digital pH meter.

Rumen fluid samples were filtered through four layers of cheesecloth to remove particulate matter. Filtered samples were divided into subsamples for the determination of ammonia nitrogen (NH₃-N), total volatile fatty acid (VFA) concentration, and protozoa counts. Samples designated for NH₃-N analysis were acidified with concentrated sulfuric acid and stored under refrigerated conditions until analysis.

Rumen ammonia nitrogen concentration (mg/L) was determined using the Kjeldahl distillation method. Total volatile fatty acid concentration was determined by steam distillation. Protozoa counts were performed using a light microscope and a counting chamber following standard procedures. Sampling time was standardized across all treatments to minimize diurnal variation in rumen fermentation parameters and to allow valid comparisons among dietary groups.

### Chemical analyses

Feed, refusal, and fecal samples were analyzed for dry matter, crude protein, ether extract, and ash according to AOAC procedures. Neutral detergent fiber and acid detergent fiber contents were determined using the detergent fiber system described by Van Soest et al. Metabolizable energy (ME) values of the diets were calculated according to the prediction equations described by the Turkish Standards Institute (TSE 1991) based on the chemical composition of the feeds.

### Statistical analysis

Data parameters evaluated at a single time point (apparent nutrient digestibility coefficients, rumen fermentation characteristics, and slaughter and carcass traits) were analyzed using one-way analysis of variance (ANOVA) via the GLM procedure, with dietary treatment as the fixed effect. When a significant treatment effect was detected, differences among means were compared using Tukey’s multiple comparison test. Conversely, growth performance parameters measured repeatedly over time—including body weight (BW), dry matter intake (DMI), average daily gain (ADG), and feed conversion ratio (FCR)—were analyzed using a linear mixed-effects model for repeated measures. The statistical model included dietary treatment (group), time (days/periods), and their interaction (Group x Time) as fixed effects, with the individual lamb as the experimental unit and included as a random effect. Model parameters were estimated using the restricted maximum likelihood (REML) method with Type III tests for fixed effects. Appropriate covariance structures were selected based on the best model fit for each parameter: heterogeneous first-order autoregressive ARH(1) for DMI, first-order autoregressive AR(1) for BW and ADG, and diagonal (DIAG) for FCR. Estimated marginal means were compared using a Bonferroni adjustment, with significance set at *P* < 0.05. All results are presented as means or estimated marginal means with the standard error of the mean (SEM) reported.

## Results

### Growth performance and feed intake

Body weight changes and growth performance parameters are presented in Tables 3 and 4. Body weight increased progressively throughout the experimental period in all treatment groups (*P* < 0.001), indicating normal growth of lambs under intensive feeding conditions. However, neither dietary treatment nor the treatment × time interaction significantly affected body weight throughout the experimental period (*P* > 0.05).

**Table 3:** Body weight changes (kg) and mixed model fixed effects across chromium picolinate and bicarbonate treatment groups

**Time (day)**	**Control**	**CrPic**	**Bic**	**CrPic + Bic**
0	26.014	25.414	25.786	25.871
14	29.086	28.457	28.014	28.586
28	31.729	31.186	30.957	31.571
42	35.629	34.086	35.000	35.371
56	40.343	38.171	37.786	38.871
63	42.471	39.829	40.371	40.857
**Type III tests of fixed effects**				
**Source**	**F**		**P-value**	
G	0.485		0.696	
T	260.237		< 0.001	
G × T	1.440		0.140	

* Values represent estimated marginal means ± standard error of the mean (SEM). SEM was 0.969 for all interaction cells. G: Group effect; T: Time effect; G × T: Group × Time interaction. P-values were derived from Type III tests of fixed effects. Models were fitted using restricted maximum likelihood (REML) estimation with appropriate covariance structures selected based on model fit for each parameter. CrPic: Chromium Picolinate; Bic: Bicarbonate; CrPic + Bic: Chromium plus Bicarbonate. Total number of animals: *n* = 28

Dry matter intake (DMI) was not significantly influenced by dietary treatment (*P* = 0.214), although a significant effect of time was observed (*P* < 0.001). The treatment × time interaction for DMI was not significant (*P* = 0.055), indicating that feed intake patterns were generally comparable among groups throughout the study.

Average daily gain (ADG) was significantly affected by dietary treatment (*P* = 0.022), time (*P* < 0.001), and the treatment × time interaction (*P* = 0.003). Lambs receiving the combined chromium picolinate and sodium bicarbonate supplementation exhibited the greatest ADG during the 14–28 day period, whereas treatment responses varied across subsequent growth phases.

Feed conversion ratio (FCR) was also influenced by dietary treatment (*P* = 0.020), time (*P* < 0.001), and the treatment × time interaction (*P* = 0.004). The most favorable feed efficiency was observed in the CrPic + Bic group during the early growth period (days 14–28), while treatment differences became less consistent during later stages of the experiment.

### Apparent nutrient digestibility

Apparent nutrient digestibility coefficients are summarized in Table 4. Digestibility of dry matter, organic matter, crude protein, and crude fat did not differ significantly among dietary treatments (*P* > 0.05; Table 4).

In contrast, fiber digestibility responses varied according to dietary supplementation. Neutral detergent fiber digestibility tended to be higher in lambs receiving chromium picolinate compared with the control group (*P* = 0.067; Table 4), while acid detergent fiber digestibility was significantly improved by chromium picolinate supplementation (*P* < 0.05; Table 4). Sodium bicarbonate supplementation, either alone or in combination with chromium picolinate, did not result in additional improvements beyond chromium picolinate alone.

**Table 4:** Dry matter intake. average daily gain. and feed conversion ratio of animals in response to dietary chromium picolinate and bicarbonate supplementation*

**Dry Matter Intake (g)**					
**Time (Days)**	**Control**	**CrPic**	**Bic**	**CrPic + Bic**	**SEM**
0–14	1126.07	1214.28	1083.31	1152.38	51.97
14–28	1463.28	1472.43	1380.96	1433.85	31.91
28–42	1688.74	1668.44	1692.93	1682.48	89.75
42–56	1741.49	1718.37	1697.86	1741.90	23.09
56–63	1930.25	1926.19	1898.69	1912.34	10.05
**Type III tests of fixed effects**					
**Source**	**F**		**P-value**		
G	2.568		0.214		
T	561.682		< 0.001		
G × T	1.441		0.055		
**Average Daily Gain (g)**					
0–14	251.02	244.90	215.31	217.35	11.33
14–28	207.14	198.98	236.73	255.10	11.44
28–42	267.35	218.37	266.33	257.14	15.81
42–56	331.63	269.39	265.31	255.10	16.44
56–63	287.76	271.43	269.39	273.47	16.15
**Type III tests of fixed effects**					
**Source**	**F**		**P-value**		
G	3.336		0.022		
T	12.897		< 0.001		
G × T	3.104		0.003		
**Feed Conversion Ratio**					
0–14	4.510	4.990	5.080	5.330	0.236
14–28	7.130	7.470	5.980	5.670	0.321
28–42	6.460	7.770	6.550	6.740	0.448
42–56	5.290	6.550	6.520	7.040	0.394
56–63	6.840	7.250	7.330	7.090	0.471
**Type III tests of fixed effects**					
**Source**	**F**		**P-value**		
G	3.417		0.020		
T	32.182		< 0.001		
G × T	2.972		0.004		

*Values are presented as estimated marginal means ± standard error of the mean (SEM). G: group effect; T: time effect; G × T: group × time interaction. P-values were obtained from Type III tests of fixed effects using mixed models with restricted maximum likelihood (REML) estimation and covariance structures selected according to the best fit for each parameter: autoregressive heterogeneous ARH(1) for Dry Matter Intake (DMI), first-order autoregressive [AR(1)] for Average Daily Gain (ADG), and diagonal covariance structure for Feed Conversion Ratio (FCR). CrPic: chromium; Bic: bicarbonate; CrPic + Bic: chromium plus bicarbonate. Total number of animals: *n* = 28

### Rumen fermentation characteristics

Rumen fermentation parameters are presented in Table 5. Mean rumen pH values did not differ significantly among treatments (*P* > 0.05; Table 5). Similarly, total volatile fatty acid concentrations were comparable across dietary treatments (*P* > 0.05; Table 5).

In contrast, ruminal ammonia nitrogen concentration was markedly affected by dietary supplementation. The highest NH₃-N values were observed in the control group, whereas lambs receiving chromium picolinate exhibited significantly lower concentrations (*P* < 0.001; Table 5). Protozoal population density also differed among treatments, with the highest counts recorded in lambs supplemented with chromium picolinate (*P* < 0.001; Table 5).

**Table 5:** Rumen fermentation characteristics of lambs fed experimental diets

Item	Control	CrPic	Bic	CrPic + Bic	SEM	*P*-value
pH	6.03	6.08	6.24	6.12	0.07	0.401
TVFA	97.94	87.70	94.86	89.37	4.50	0.357
NH3-N	226.29a	168.28b	198.57a	138.71b	10.30	< 0.001
Protozoa	674857b	906571a	499714c	599143bc	40,000	< 0.001

CrPic: chromium picolinate; Bic: sodium bicarbonate; NH₃-N: ammonia nitrogen (mg/L); TVFA: total volatile fatty acids (mmol/L); SEM: standard error of the mean. Each lamb was considered the experimental unit. Means within a row with different superscripts differ significantly (*P* < 0.05). Protozoa counts are expressed as cells ×10³/mL of rumen fluid

### Slaughter traits and carcass characteristics

Slaughter weight, hot carcass weight, cold carcass weight, and dressing percentage are presented in Table 6. No significant differences were detected among dietary treatments for slaughter weight or carcass yield parameters (*P* > 0.05; Table 6).

External carcass measurements, backfat thickness, and the area of the Musculus longissimus dorsi were also not influenced by dietary supplementation (*P* > 0.05; Table 6). However, skin weight and testicular weight differed among treatments, with lower values observed in lambs receiving sodium bicarbonate- and chromium-containing diets compared with the control group (*P* < 0.05; Table 6).

**Table 6:** Slaughter traits and carcass characteristics of lambs fed experimental diets

Item	Control	SEM	CrPic	SEM	Bic	SEM	CrPic + Bic	SEM	*P*-value
Slaughter weight (kg)	42.47	0.735	39.83	1.070	40.37	0.872	40.86	1.257	0.3002
Hot carcass weight (kg)	18.99	0.323	18.15	0.575	18.16	0.617	18.53	0.592	0.6546
Cold carcass weight (kg)	18.52	0.318	17.66	0.583	17.72	0.616	18.07	0.627	0.6811
Hot carcass yield (%)	44.72	0.312	45.53	0.418	44.91	0.697	45.36	0.475	0.6347
Cold carcass yield (%)	43.60	0.308	44.31	0.473	43.83	0.717	44.22	0.542	0.7591
Cooling loss (%)	2.51	0.129	2.69	0.184	2.40	0.010	2.52	0.380	0.8435
MLD subcutaneous fat thickness (mm)	3.55	0.232	3.47	0.095	3.76	0.172	3.68	0.174	0.6512
MLD area (cm²)	13.53	1.06	13.25	0.69	13.84	0.34	13.49	0.79	0.9589
Skin weight (kg)	4.63	0.139	4.09	0.139	3.97	0.139	4.53	0.139	0.0170
Testes weight (g)	416.43	23.369	346.43	23.369	345.00	23.369	310.71	23.369	0.0175

CrPic: chromium picolinate; Bic: sodium bicarbonate; MLD: Musculus longissimus dorsi; SEM: standard error of the mean. P-values are from one-way ANOVA

## Discussion

The present study provides a detailed evaluation of the individual and combined effects of chromium picolinate and sodium bicarbonate supplementation in lambs fed high-concentrate diets, with particular emphasis on growth performance, rumen fermentation, nutrient digestibility, and carcass characteristics. By integrating metabolic modulation and ruminal buffering strategies within a controlled experimental design, this study addresses a critical gap in the literature regarding the combined application of these additives under intensive lamb-fattening conditions.

### Growth performance and feed efficiency in the context of high-concentrate feeding

High-concentrate feeding systems are widely adopted to enhance growth rates and economic efficiency in lamb production; however, their effects on growth performance are often inconsistent due to interactions between diet composition, rumen function, and post-absorptive metabolism (Plaizier et al. 2018; Humer et al. 2018; Zebeli and Metzler-Zebeli 2019; Ding et al. 2020). In the present study, overall dry matter intake and final body weight were not significantly affected by dietary treatments, indicating that neither chromium picolinate nor sodium bicarbonate adversely influenced feed palatability or voluntary intake. Similar findings have been reported in lambs and growing ruminants receiving organic chromium or buffering agents under nutritionally adequate conditions (Penner et al. 2019; McCann et al. 2020; López et al. 2022; Eun et al. 2022).

Despite the absence of sustained effects on final body weight, lambs receiving combined chromium picolinate and sodium bicarbonate supplementation exhibited improved feed efficiency during the early growth phase. Phase-dependent responses to dietary additives have been previously described, particularly during periods of metabolic adaptation to high-energy diets (Qiu et al. 2022; Bai et al. 2023; Hung et al. 2023). These transient improvements may reflect enhanced efficiency of energy utilization rather than increased nutrient intake, consistent with the role of chromium in improving insulin sensitivity and glucose metabolism (Spears et al. 2017; Vincent 2019; Sun et al. 2021).

The lack of persistent growth responses over the entire experimental period aligns with reports suggesting that chromium supplementation does not universally enhance average daily gain but may exert context-specific effects depending on physiological stage, dietary challenge, and metabolic status (Ren et al. 2020; Bai et al. 2021; Qiu et al. 2022). Collectively, these findings suggest that the benefits of combined chromium picolinate and sodium bicarbonate supplementation may be most pronounced during early adaptation to high-concentrate feeding rather than during steady-state growth.

### Apparent nutrient digestibility and implications for rumen function

Apparent digestibility of dry matter, organic matter, and crude protein was not markedly influenced by dietary treatments, indicating that basal nutrient utilization remained stable across experimental groups. This observation is consistent with previous studies reporting limited effects of chromium or buffering agents on overall digestibility when diets are formulated to meet nutrient requirements (Dong et al. 2019; Carmona et al. 2022; Oliveira et al. 2023). However, the significant improvement in acid detergent fiber digestibility observed with chromium picolinate supplementation highlights a selective effect on fiber utilization.

Although chromium is not traditionally considered a rumen-active element, emerging evidence suggests that chromium supplementation may indirectly influence rumen microbial efficiency by altering fermentation end-products and energy partitioning (Reynolds et al. 2019; Khiaosa-ard and Zebeli 2019; Li et al. 2021). Increased propionate production associated with chromium supplementation has been linked to enhanced microbial growth efficiency and improved fiber degradation under certain conditions (Patra and Aschenbach 2018; Hristov et al. 2019; Li et al. 2021). The selective improvement in fiber digestibility observed in the present study may therefore reflect enhanced microbial utilization of structural carbohydrates rather than generalized improvements in digestion.

In contrast, sodium bicarbonate supplementation did not result in additional improvements in fiber digestibility beyond those observed with chromium picolinate alone. Buffering agents are known to enhance fiber digestion primarily under conditions of pronounced ruminal acidification (Gallo et al. 2020; Eun et al. 2022; Carmona et al. 2022). The relatively stable rumen pH observed across treatments in the present study likely limited the potential impact of sodium bicarbonate on fiber degradation, underscoring the importance of baseline rumen conditions in determining additive efficacy (Penner et al. 2019; Oliveira et al. 2023).

### Rumen fermentation and nitrogen metabolism as key response variables

Rumen fermentation characteristics provide critical insight into the mechanisms underlying treatment effects. Mean rumen pH and total volatile fatty acid concentrations were not significantly altered by dietary supplementation, indicating that the high-concentrate diet employed did not induce severe ruminal acidification. Similar findings have been reported in studies where buffering agents exerted limited effects on rumen pH under moderately controlled feeding conditions (Gallo et al. 2020; López et al. 2022; Oliveira et al. 2023).

In contrast, ruminal ammonia nitrogen concentration was markedly reduced in lambs receiving chromium picolinate supplementation, suggesting improved nitrogen utilization efficiency. Lower ruminal ammonia concentrations are commonly associated with enhanced microbial protein synthesis and reduced nitrogen losses (Patra and Aschenbach 2018; Hristov et al. 2019; Morgavi et al. 2020). Similar reductions in ruminal ammonia following chromium supplementation have been reported in cattle and sheep, supporting a potential role of chromium in modulating microbial nitrogen assimilation (Spears 2019; Sano et al. 2019; Bai et al. 2021; Zhang et al. 2024).

The increase in protozoal population density observed in chromium-supplemented lambs further suggests altered rumen microbial dynamics. While protozoa are often associated with increased ammonia production due to bacterial predation, their role in rumen ecology is complex and context-dependent (Khiaosa-ard and Zebeli 2019; Morgavi et al. 2020). Increased protozoal activity may reflect a more stable rumen environment and enhanced microbial turnover, potentially contributing to improved nitrogen utilization under high-concentrate feeding conditions. Although protozoal population density was higher in chromium-supplemented lambs, the concurrent reduction in ruminal ammonia-N concentration suggests improved microbial nitrogen capture and reduced proteolysis, indicating a shift toward more efficient nitrogen utilization rather than increased deamination.

Sodium bicarbonate supplementation resulted in intermediate ammonia nitrogen concentrations, indicating a modest stabilizing effect on rumen nitrogen metabolism without directly enhancing microbial efficiency. This finding aligns with previous studies suggesting that buffering agents primarily influence physicochemical conditions in the rumen rather than directly modulating nitrogen metabolism (Penner et al. 2019; Carmona et al. 2022). This apparent paradox suggests that chromium supplementation may have enhanced microbial nitrogen capture efficiency, limiting net ammonia accumulation despite increased protozoal density. The lack of a significant pH response likely reflects the absence of severe ruminal acidification under the controlled feeding conditions applied in this study.

### Carcass traits and translation of metabolic responses

Despite measurable effects on rumen fermentation and nutrient utilization, dietary supplementation did not significantly influence major carcass characteristics, including dressing percentage and longissimus dorsi muscle area. This lack of response suggests that the observed metabolic and fermentative changes were insufficient in magnitude or duration to translate into enhanced tissue accretion. Similar outcomes have been reported in studies evaluating chromium supplementation in lambs and cattle, where improvements in metabolic efficiency did not consistently result in altered carcass traits (Ren et al. 2020; Bai et al. 2021; Qiu et al. 2022).

Differences observed in skin and testicular weights should be interpreted cautiously, as these parameters are not directly related to carcass value and may reflect treatment-related variation in tissue distribution rather than economically relevant changes. The limited impact of supplementation on carcass traits underscores the importance of evaluating additive strategies within the broader context of production objectives and economic returns. The inclusion of carcass traits aimed to evaluate whether metabolic responses translated into commercially relevant outcomes, despite the absence of significant effects.

### Integrated interpretation and implications for intensive lamb production

Collectively, these findings indicate that chromium picolinate and sodium bicarbonate exert complementary but distinct physiological effects in lambs fed high-concentrate diets. Chromium supplementation primarily influenced nitrogen metabolism and fiber digestibility, whereas sodium bicarbonate contributed to maintaining ruminal stability. The combined supplementation strategy improved feed efficiency during specific growth phases, suggesting that its benefits may be most pronounced during adaptation to intensive feeding systems. However, the absence of consistent improvements in final body weight and carcass characteristics indicates that the effectiveness of this strategy depends on dietary conditions, animal physiology, and the duration of supplementation. Future studies should further investigate optimal supplementation protocols and the metabolic mechanisms underlying these responses.

## Conclusions

Chromium picolinate and sodium bicarbonate supplementation exerted distinct physiological effects on rumen fermentation characteristics and nutrient utilization in lambs fed high-concentrate diets. Chromium picolinate primarily influenced rumen nitrogen metabolism and fiber digestibility, as evidenced by reduced ruminal ammonia nitrogen concentration and improved acid detergent fiber digestibility, whereas sodium bicarbonate contributed to stabilization of rumen fermentation without markedly altering intake or overall digestibility.

The combined supplementation strategy resulted in phase-dependent improvements in feed efficiency during the early stages of the fattening period, although these responses were not consistently reflected in all carcass characteristics.

## Data Availability

The datasets generated and/or analyzed during the current study are available from the corresponding author on reasonable request.
